# Inverse design of lateral hybrid metasurfaces structural colour: an AI approach†

**DOI:** 10.1039/d4ra04981k

**Published:** 2024-08-15

**Authors:** Rui Fang, Amir Ghasemi, Dagou Zeze, Mehdi Keshavarz Hedayati

**Affiliations:** a Department of Engineering, Durham University Durham DH1 3LE UK mehdi.keshavarz-hedayati@durham.ac.uk

## Abstract

In conventional metasurface structural colour design, simulations combined with human intuition are used for design and optimization, making it challenging to find the best solution. Here we introduce an innovative AI-assisted design process that bypasses the need for complex simulations, enabling swift and precise mapping between metasurface parameters and colour coordinates. Instead of assigning one colour to one geometry, we demonstrate that multiple colours can be generated from a single geometry under varying levels of strain. This can be achieved through a single model, facilitating the development of active metasurfaces using AI. This finding enables designers to create active metasurfaces that account for both geometric properties and dynamic responses in a unified model which could accelerate the development of active metamaterials closer to practical applications in the real world.

## Abstract 2

In this work, we employ machine learning as a tool to optimise the design process, enabling the identification of the most effective metasurface structural colour without relying on conventional trial-and-error methods.

## Graphical abstract

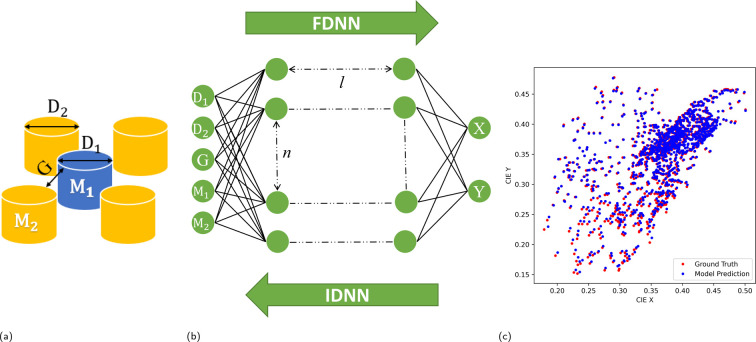

## 1 Introduction

Optical metasurfaces, intricate 2D structures influencing light–matter interaction at a subwavelength scale, generate structural colours (SC) through the interaction of light with periodic nano-resonators (NRs). These resonators, often composed of noble metals^1^ and high refractive index dielectrics,^2^ exhibit favorable optical properties. The plasmonic resonance of metallic NRs can create an intense field enhancement in the gap region, they are naturally sensitive to the change in the surrounding environment.^3,4^ The high permittivity, low-loss dielectrics have the capacity to generate robust magnetic resonance within the NR through Mie resonance.^4^ A hybrid system composed of both metal and dielectric can instead benefit from both worlds.

Active metasurfaces have emerged as transformative technologies for real-time control over electromagnetic waves,^5^ promising applications in beam steering,^6,7^ sensing,^8,9^ and communication.^10^ Stimuli for active metamaterials encompass various categories, including mechanical,^11^ optical and magnetic reconfiguration by using active molecules,^7,12^ magnetically tunable elements,^13^ and thermally responsive materials.^14^

Mechanically active metasurfaces have attracted significant attention from researchers due to their ease of handling and potential for mass fabrication. However, these stretchable metamaterials face significant challenges, one of which is the mismatch in surface tension between the flexible polymer substrate and the rigid resonating materials. This disparity can lead to the rigid material cracking after a single stretch, thereby impeding its ability to achieve the desired colour tuning range and without compromising its intended optical functions.^15^ Our recent breakthrough introduces a groundbreaking concept – the ‘lateral hybrid metasurface’.^9^ Unlike conventional layering hybrid design,^16,17^ this mechanically tunable metamaterial arranges metal and dielectric resonators in a lattice formation, demonstrating high sensitivity to mechanical forces. Large tunability covering 14% of the sRGB colour map has been observed across the full range of colour with only a 10% strain.^9^ This lateral hybrid system, showcased for the first time, exhibits simplicity in creating a reversible, highly sensitive, and power-efficient tunable SC metasurface.

Another challenge faced by stretchable metasurface is the complexity of their design process. Currently, designing these metasurfaces involves a cumbersome procedure that starts from parameters and ends with colours. This method relies on time-consuming numerical simulations and human intuition, making it difficult to achieve global optimization. Deep Learning (DL), a subset of machine learning (ML), offers a paradigm shift in metasurface design. Unlike the computationally intensive finite element method (FEM) or finite-difference time-domain (FDTD) processes,^18–20^ DL leverages multi-layer neural networks for efficient feature extraction and data learning.^21^ In the conventional design process, where parameters are tuned to achieve a specific colour, local optimization relies on subjective human estimations, making it challenging to reach a global optimum. Inverse design uncovers hidden correlations between parameters and reflected colour without the need to solve complex equations. Utilizing DL, we shift from the traditional parameter-to-colour method to a colour-to-parameter approach, overcoming the limitations of human estimations and achieving global optimization.

While some studies have used AI to reverse the design flow from colours to parameters, the data provided typically consists of a single-colour point, or the tuning effect relies on changes in resonator geometry.^19,22,23^ As a result, the true tuning effect in stretchable SCs has yet to be fully realized. Our DL approach automates the design process, predicting not only single-coordinate for a given colour, but also one geometry with varying strain levels given a range of colour. This significantly decreases computational time compared to traditional methods such as FEM and allows for the prediction of stretchable metasurface structures for any set of colours. This innovation holds promise for efficient and precise metasurface design.

## 2 Material and method

**Fig. 1:**
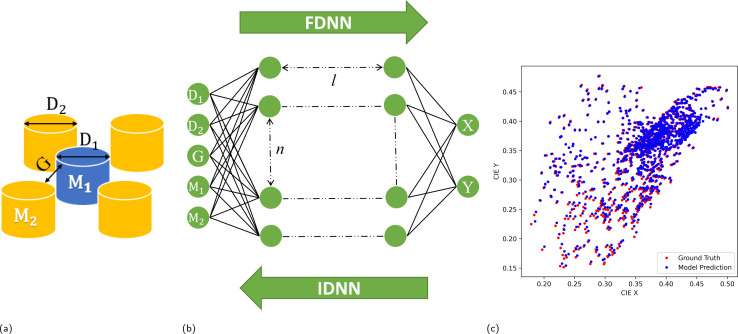
(a) The structure of metasurface composed of metal and dielectric resonators where blue and yellow represent dielectric and metal NRs respectively. Materials, gap, diameter 1 and 2 are marked as M_1_, M_2_, *G*, *D*_1_, *D*_2_ respectively. (b) Fully connected DNN, where *l* and *n* are the number of hidden layers and neurons in each hidden layer, respectively. FDNN denotes forward design neural network and IDNN for inverse design neural network. (c) The colour coordinate from the validating dataset is shown, where red points are ground truth (input data) and blue points are model prediction.

Fig. 1 illustrates the general design process. The composition of the metasurface (Fig. 1a) is processed through a DL model (Fig. 1b), and its corresponding colour is matched and visualized in the CIE colour diagram (Fig. 1c). A typical lateral hybrid metasurface is presented in Fig. 1a, where blue and yellow pillars represent dielectric and metal resonators, respectively. Note that, M_1_, M_2_ stands for the type of the materials, *G* represents the inter-particle gap and dielectric and metal pillar diameters are shown as *D*_1_, and *D*_2_, respectively.

There are various choices in metals and dielectric materials with intermediate or high refractive indices. To diversify the response of the active metamaterial, we have chosen a range of commonly used materials as listed in Table 1. The chosen metals and dielectrics are Ag, Al, Au, Cu, Li, Ti and Al_2_O_3_, GaAs, GaSb, Ge, ITO, Si, Si_3_N_4_, TiO_2_, and ZnO. These materials were selected based on their extensive study in the literature, ensuring a wealth of ESI data,† and their proven accessibility and compatibility with standard laboratory equipment, facilitating efficient fabrication and experimental validation.^22,24,25^

**Table 1:** DL training parameters for two models: single-coordinate prediction and tuning prediction. Both models utilize the same materials and similar diameter ranges. However, the key distinction lies in the parameter for the gap size (*G*). In the single-coordinate prediction model, *G* ranges from 0.5 nm to 50 nm, while in the tuning prediction model, *G* is fixed at five predetermined values as shown below

Parameter	Single	Tuning
*D*_1_	58–174 nm	56–176 nm
*D*_2_	40–164 nm	38–166 nm
*G*	0.5–50 nm	2, 4, 8, 14, 22 nm
M_1_	Al_2_O_3_, GaAs, GaSb, Ge, ITO, Si, Si_3_N_4_, TiO_2_, ZnO	
M_1_	Ag, Al, Au, Cu, Li, Ti	
Total number	4128	14 668

These materials exhibit excellent optical properties crucial for generating high-quality structural colours (SCs). Metals like Ag, Al, Au, and Cu are known for their plasmonic properties, enabling strong light interaction and intense field enhancement,^1,26^ while high refractive index dielectrics like Al_2_O_3_, GaAs, GaSb, Ge, ITO, Si, Si_3_N_4_, TiO_2_, and ZnO support strong Mie resonances with low-loss characteristics.^2,27^ This combination allows for a broad range of optical responses, making our metasurfaces versatile and high-performing, with diverse and tunable structural colour properties.

This table also presents data composition used in both single and tuning scenarios. For parameters *D*_1_ and *D*_2_, the ranges differ slightly between the two scenarios. In single, *D*_1_ ranges from 58 to 174 nm, while in tuning, it's 56 to 176 nm. Similarly, for *D*_2_, the ranges are 40 to 164 nm in single and 38 to 166 nm in tuning. Parameter *G* varies from 0.5 to 50 nm in single and includes specific values (2, 4, 8, 14, and 22 nm) in tuning. The total number of cases is 4128 in single and 14 668 in tuning.

COMSOL Multiphysics simulation is adapted to generate the reflectance of the designed structures. The corresponding colour coordinate is calculated to derive the CIE1931 RGB spectral chromatic coordinates.^28^

First, we examine the ability to design a set of parameters based on a single colour coordinate. Then to mimic a tuning effect, 5 coordinates have been used to predict a design which includes identical materials and diameters, but for 5 different strain levels (gap sizes). The model's hyperparameters are informed by previous studies,^19,29,30^ with specific settings including the choice of loss function (Mean Absolute Error – MAE), a learning rate of 0.15, and the activation function being Leaky ReLU. Additionally, the Adam optimizer is employed, and a dropout rate of 0.1 is applied to each layer to mitigate overfitting risks. To determine the optimal network structure, a systematic sweep of neuron layer (3–10) and depth (3–1500) configurations has been conducted. The findings of the sweep are detailed in the subsequent sections of this report.

**Fig. 2:**
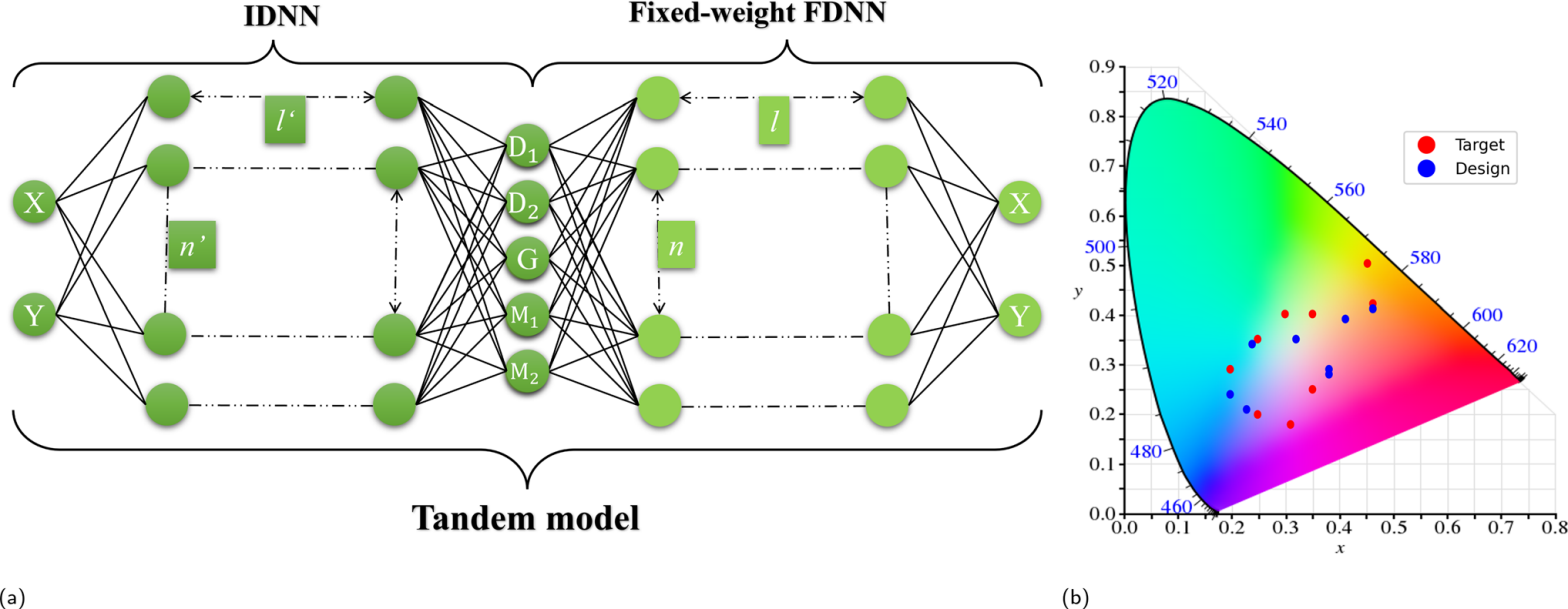
(a) Fully connected tandem model where an IDNN is added in front of a fixed-weight FDNN. (b) Validation data set shown on a CIE diagram, where red points are ground truth (input data) and blue points are model prediction.

The inverse correspondence between electromagnetic (EM) response and SC is characterized by a one-to-many relationship, indicating that numerous metamaterial configurations could produce identical SCs. It is noteworthy that DNNs, being one-to-one nonlinear models, are inherently limited in their capacity to directly acquire the correlation, in contrast to the forward relationship.^19,31^ To overcome the issue, a tandem auto-encoder architecture is employed in the single-colour design. Fig. 2 shows a diagram of the learning architecture and its predicting accuracy. In 2a the pre-trained forward deep neural network (FDNN) model is joined with an inverse design neural network (IDNN) sharing one hidden layer. The hidden layer joining the two networks represents the latent encoding of geometry parameters *D*_1_, *D*_2_, *G*, M_1_ and M_2_.

Three parameters are employed for the assessment of a model's performance: training accuracy, validation accuracy and design accuracy. Training and validation accuracy is characterized by the complement of the Mean Absolute Error (1-MAE) based on training and validation data during the training process. Design accuracy describes the ability of a model to accurately design parameters when provided with new data. It is determined by the discrepancy between the designed and the target coordinate.

## 3 Results and discussion

### 3.1 Single coordinate prediction *via* inverse design

A scheme of the network structure which is adapted to predict a set of geometry (*D*_1_, *D*_2_, M_1_, M_2_ and *G*) from a single colour coordinate (*X*, *Y*) is shown in Fig. 1b. Initially, we train the FDNN independently using available data. A total number of 4128 colour coordinates and their corresponding geometries are employed in training the FDNN model, with an 80% allocated for training and the remaining 20% for validation. The validation data and their predictions by the FDNN model is shown in Fig. 1c, which are represented by red and blue dots respectively. The loss metric is defined as the MAE between the desired colour and the predicted colour.

Once the FDNN has been trained, a tandem model is constructed to prevent the aforementioned ‘one-to-many’ problem, where the fixed-weight FDNN and the IDNN are joined together sharing one hidden layer which contains the geometry information, as shown in Fig. 2a. In the tandem model, which consists of a pre-trained FDNN followed by an IDNN, the training process involves optimizing the performance of the IDNN while keeping the weights of the FDNN fixed. Essentially, the training of the tandem model can be viewed as an unsupervised training of the IDNN. During this training, the same colour coordinates are used as both input and output. The IDNN learns to find the best solution mapping from the input colour to the hidden geometry layer. Following the training process, we separate the IDNN from the tandem structure to utilize it as a standalone model for geometry prediction. This approach leverages the capabilities of both the FDNN and IDNN, with the FDNN providing initial feature extraction and the IDNN refining the prediction process based on the specific task at hand.

To determine the optimal network architecture, an exhaustive search of the network layer *l* and neuron number of each layer *n* has been conducted. The range for the number of network layers *l* span from 3 to 10, with an step of 1. Neuron numbers for all layers are set the same for easier analysis. Within each layer, the number of neurons *n* are studied in three ranges: 3 to 30 with an step of 1, 30 to 300 with an step of 10, and 300 to 1500 with an step of 100. For instance, the simplest configuration consists of 3 layers, each with 3 neurons, while the most complex setup includes 10 layers, each with 1500 neurons.

**Fig. 3:**
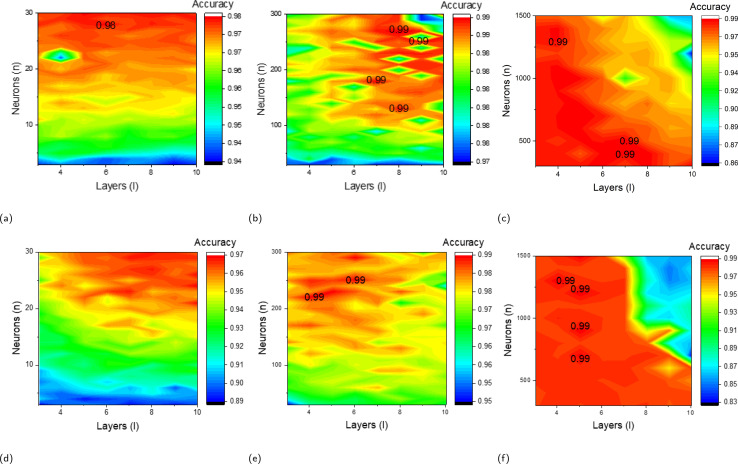
The sweeping of parameters *l* and *n* is examined. (a–c) Display the result in single colour INN models and (d–f) for multiple colour tuning models. In this context, the variable *l* is represented along the *x*-axis, spanning a range from 3 to 10 with an incremental step of one. The variable *n* is plotted on the *y*-axis, encompassing a range from 3 to 10 with a step of one (a and d), 30 to 300 with an step of 10 (b and e), and 300 to 1500 with a step of 100 (c and f). The graphs depict the highest accuracy achieved by various configurations, with labels denoting each.

In Fig. 3a–c, the validation accuracy for these configurations is presented, revealing multiple instances of peak accuracy. Ultimately, achieving the same highest accuracy of 99%, we opt for the simplest configuration with *n* = 8 and *l* = 130, offering a speed of 0.17 ms per step. This speed is notably lower than that of the other configurations, all of which exceed 0.75 ms per step. By selecting this configuration, we effectively minimize computational costs.

To assess the design accuracy of the inverse design, ten coordinates are randomly generated. The results have been depicted in Fig. 2b. The IDNN utilizes the target coordinates to generate corresponding parameters, which are then processed using COMSOL Multiphysics to derive the corresponding reflectance. Subsequently, the reflectance spectrum is then encoded into colour coordinates. The model is able to produce versatile designs given different coordinates. The designed colours exhibit a notable degree of proximity to the target colours, encompassing a broad spectrum of hues. The majority of target and designed colours show a close correspondence, although there are instances where a relatively substantial disparity is observed, as exemplified by the example around the orange colour fields in Fig. 2b. This discrepancy can be attributed to the non-uniform distribution of the CIE colour space, wherein a uniform adjustment in coordinates may lead to minimal colour variation in certain regions while causing a significant colour shift in others.^32^ Despite the nonuniform, the overall design performance yields a remarkable average design accuracy of 90%.

### 3.2 Tuning prediction

The previous model demonstrates that accurate parameters corresponding to a provided colour coordinate can be generated. Nevertheless, its capacity to generate a diverse range of parameters with specific colour tuning demand is limited. Thus, a tuning model is built where the 5 coordinates are set as the output vector while the corresponding M_1_, M_2_, *D*_1_, *D*_2_, *G*_1_–*G*_5_ are set as input. The FDNN model, trained in the preceding step, is used for data generation *in lieu* of COMSOL. This transition results in a substantial enhancement in time efficiency, representing a computational time reduction by a factor of 2^14^ times (257 s/coordinate for COMSOL simulation and 17 ms/coordinate for DNN model), while maintaining a validation accuracy rate of 99% (Fig. 3d and e). 14 668 data are generated to facilitate the training of a tuning DNN. Together with the 4128 original data, a total number of 18 796 data has been used to train the tuning model. To simulate different strain levels, the geometric and material parameters remain fixed, and only the gap size has been systematically increased to 2 nm, 4 nm, 8 nm, 14 nm and 22 nm. In contrast to the preceding case, where single-coordinate prediction has been the method employed, the tuning problem exhibits a distinctive characteristic stemming from the heightened complexity inherent in the input data. From a statistical standpoint, it is exceedingly improbable for a set of coordinates to yield multiple distinct designs. As a result, the tuning model is less likely to be confronted with the one-to-many mapping challenge, and, consequently, there is no necessity for the implementation of a tandem model.

The validation accuracy of the *n* and *l* sweep can be found in Fig. 3d–f. The graph illustrates the highest accuracy of 99% has been achieved at several places. For computation simplicity, we select the structure that contains the lowest number of *n* and *l*, where *l* = 5 and *n* = 230.

Based on our previous experience with stretching SCs, we observe a consistent trend among all colour coordinates. Specifically, we note that they all follow a smooth curve rather than zigzagging. Following a similar process as before, we generate 10 sets of colour coordinates representing the entire colour spectrum, each comprising 5 points forming a smooth curve on the CIE diagram. These sets, unfamiliar to the model, have been then inputted for parameter prediction. Subsequently, COMSOL Multiphysics has been utilized to calculate the electromagnetic (EM) response based on the predicted parameters, serving as a validation step. In Table 2, the predicted parameters are presented alongside the target design colour and the corresponding accuracy. This comprehensive analysis attests to the model's effectiveness in creating a metasurface capable of colour alteration, achieving an impressive design accuracy rate of up to 97%.

**Table 2:** Validation result of a tuning model. This table shows a list of predicted parameters given target colour coordinate and the FEM simulated design colour. Their accuracy is shows on the last column

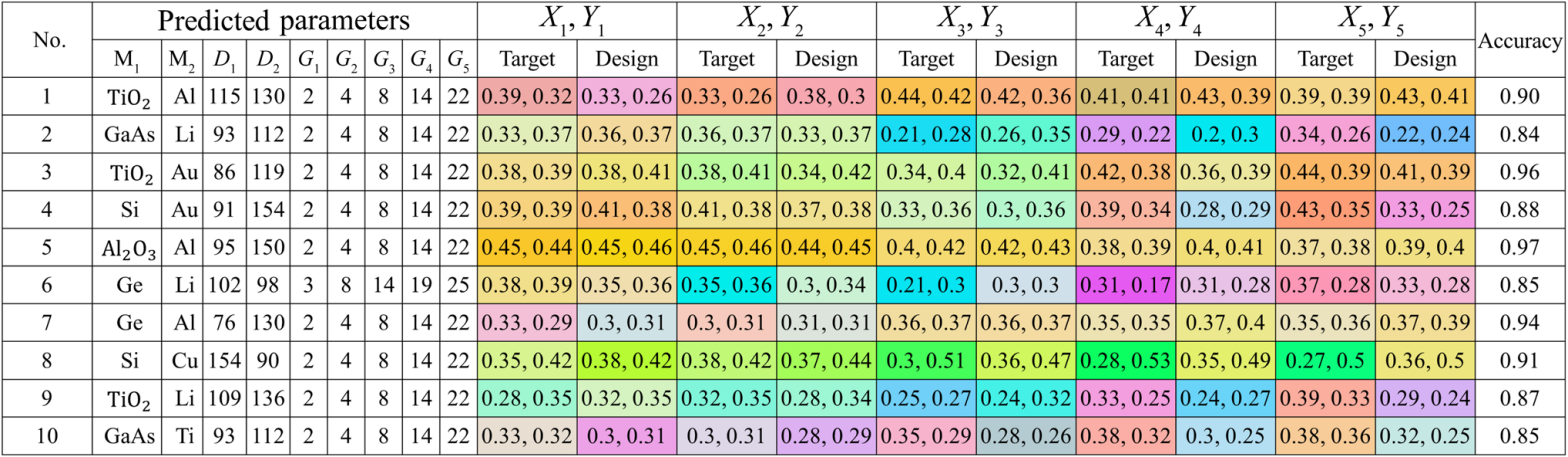

**Fig. 4:**
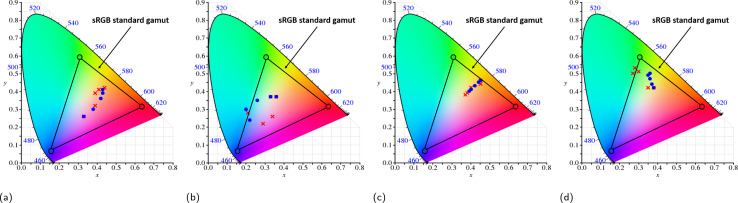
Comparison of predicted and target colour in the CIE diagram corresponding to no. 1, 2, 5 and 8 from Table 2, where red crosses and blue dots represent predicted and target colour respectively. The colour tuning ranges from (a) red to purple, (b) red to green to blue, (c) orange to red, and (d) green to red, respectively.

Further visual representations of target and design colours on a CIE diagram are presented in Fig. 4a–d, where red crosses and blue dots denote target and design colours, respectively. These diagrams correspond to no. 1, 2, 5, and 8 from Table 2, respectively. The colour range spans red, green, and blue regions, with the highest accuracy reaching 97%. Achieved tuning ranges include red to purple (Fig. 4a), red to green to blue (Fig. 4b), orange to red (Fig. 4c), and green to red (Fig. 4d). The colour scheme across the entire sRGB standard gamut underscores the model's capability to predict colour tuning across the full spectrum with high accuracy, a feat not realized by previous studies.

To add to the previously discussed CIE uniformity, the non-uniform distribution of the CIE colour space significantly affects our deep learning model's accuracy. In the single coordinate model, a noticeable colour discrepancy around the orange region on the CIE diagram can be attributed to this non-uniformity. A uniform adjustment in coordinates may lead to minimal colour variation in some regions while causing a significant colour shift in others.^32^ Although the target and designed colours appear distant on the CIE diagram, they still share a similar actual colour. In other words, the single coordinate prediction showed a significant difference in colour coordinates while the actual colours appeared quite similar.

In contrast, for the tuning prediction, the opposite problem has been observed. Large colour differences could be seen even when their colour coordinates are quite similar. For instance, in coordinate no. 9, *X*_3_, *Y*_3_, the target and designed colours are (0.25, 0.27) and (0.24, 0.32), respectively. A mere shift of (−0.01, 0.03) in the coordinates resulted in the target colour being ball blue and the designed colour aqua. Similar trends have been observed in coordinates no. 2, *X*_1_, *Y*_1_, and no. 7, *X*_2_, *Y*_2_, which are situated on the boundary of colour shifting in the CIE diagram. Despite these issues, the model managed to achieve an accuracy as high as 97%.

The successful prediction of colour tuning marks a significant step towards automating the design of structural colours using metasurfaces. This advancement holds great potential for enhancing flexibility and adaptability in this field. It also effectively addresses a challenge seen in earlier research, where colour tuning enabled by deep learning was achieved through alterations in nano-resonators’ geometries or layer thicknesses.^19,22,23^ This achievement stands as a pioneering example of predicting changes in structural colours in the context of actively tunable metasurfaces.

## 4 Conclusions

This paper presents a DL method for designing lateral hybrid metasurfaces. Unlike traditional simulation methods that go from parameters to colour, our approach reverses the process and allows the design flow to go from colour to parameters. This approach frees the design from subjective guessing, reduces computational complexity and enables automated design for tuning colours. Initially, we introduce a single-colour prediction with an impressive 90% accuracy. Furthermore, we present the parameter design process given arbitrary colour range on the CIE space. Our model achieves predicting a stretchable SC metasurface to match the given colour range with a design accuracy of up to 97%, overcoming the limitations of previous literature, which could only predict a single colour. This advancement not only enhances the potential of ultra-flexible and stretchable lateral hybrid systems but also accelerates the development of active metamaterials towards practical applications in real-world scenarios.

## Data availability

Data for this article, including COMSOL data used to train the model and the code for the models are available at Github at https://github.com/RiRiRui/MLdata.

## Conflicts of interest

There are no conflicts to declare.

## Supplementary Material

RA-014-D4RA04981K-s001
