# Supplementary material for: Inverse design of lateral hybrid metasurfaces structural colour: an AI approach
Source: RSC Adv. 2024 Aug 15;14(35):25678–84. doi: 10.1039/d4ra04981k (PMC11325218; doi:10.1039/d4ra04981k)
Supplement: RA-014-D4RA04981K-s001 [file RA-014-D4RA04981K-s001.pdf]

Supplementary Information (SI) for RSC Advances.
This journal is © The Royal Society of Chemistry 2024

Supplymentary Information: Inverse Design of Lateral Hybrid Metasurfaces Structural Colour: An AI approach

1. REFLECTANCE SPECTRUM OF PREDICTED PARAMETERS

This figure presents the reflectance spectra of the lateral hybrid metasurfaces calculated using COMSOL Multiphysics. The spectra are shown for different gap sizes (G = 2 nm, 4 nm, 8 nm, 14 nm, and 22 nm), demonstrating the variation in reflectance as a function of wavelength from 400 nm to 800 nm. Each subfigure ((a) through (d)) corresponds to specific metasurface configurations as listed in Table 2. Subfigure (a) shows the reflectance spectra for the configuration listed as No.1. Subfigure (b) displays the reflectance spectra for the configuration listed as No.2. Subfigure (c) illustrates the reflectance spectra for the configuration listed as No.5. Subfigure (d) presents the reflectance spectra for the configuration listed as No.8.

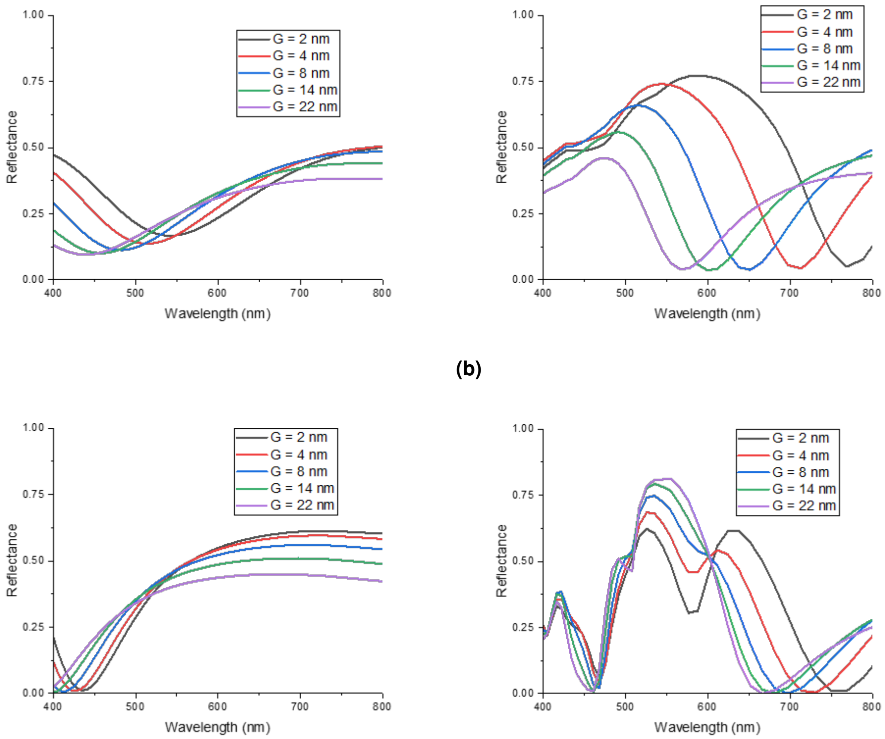

(c) (d)

Fig. S1. The reflectance spectrum calculated by COMSOL Multiphysics. (a) - (d) correspond to Fig. 4 (a) - (d) and Table.2 No.1, No.2, No,5 and No.8 respectively.

2. CIE 1931 COLOUR ENCODING

CIE 1931 XYZ tristimulus can be obtained by integrating CIE 1931 XYZ spectral tristimulus values [1]. The calculation equation is given below:

$$\begin{cases} X = k \int_{\lambda} \Phi(\lambda)\bar{x}(\lambda)\mathrm{d}\lambda \\ Y = k \int_{\lambda} \Phi(\lambda)\bar{y}(\lambda)\mathrm{d}\lambda \\ Z = k \int_{\lambda} \Phi(\lambda)\bar{z}(\lambda)\mathrm{d}\lambda \end{cases} \tag{S1}$$

where $\Phi(\lambda)$ is the spectral distribution of light stimulus and k is a normalizing constant. These integrated values $\bar{x}(\lambda)$, $\bar{y}(\lambda)$ and $\bar{z}(\lambda)$ are called the spectral tristimulus values. In the case of this paper, $\Phi(\lambda)$ should be

$$\Phi(\lambda) = E(\lambda) \cdot R(\lambda) \tag{S2}$$

where $R(\lambda)$ is the spectral reflectance of the metasurface, $E(\lambda)$ is the (relative) spectral irradiance of the illumination, and

$$k = 100 / \int_{\lambda} E(\lambda)\bar{y}(\lambda)\mathrm{d}\lambda \tag{S3}$$

By projecting the tristimulus values on to the unit plane (X+Y+Z=1), colour can be expressed in a two dimensional plane. Such a unit plane is known as the chromaticity diagram. The colour can be specified by the chromaticity coordinates (x, y) as given by:

$$\begin{cases} x = \dfrac{X}{X+Y+Z} \\ \\ y = \dfrac{Y}{X+Y+Z} \end{cases} \tag{S4}$$

REFERENCES

1. Y. Ohno, "CIE Fundamentals for Color Measurements," NIP & Digit. Fabr. Conf. **16**, 540–545 (2000).
